# Identification of a specific α-synuclein peptide (α-Syn 29-40) capable of eliciting microglial superoxide production to damage dopaminergic neurons

**DOI:** 10.1186/s12974-016-0606-7

**Published:** 2016-06-21

**Authors:** Shijun Wang, Chun-Hsien Chu, Mingri Guo, Lulu Jiang, Hui Nie, Wei Zhang, Belinda Wilson, Li Yang, Tessandra Stewart, Jau-Shyong Hong, Jing Zhang

**Affiliations:** Department of Pathology, University of Washington School of Medicine, Seattle, Washington 98104 USA; Neuropharmacology Section, Laboratory of Neurobiology, National Institute of Environmental Health Sciences National Institutes of Health, Research Triangle Park, NC 27709 USA; Institute of Molecular Medicine, National Cheng Kung University, Tainan, 70101 Taiwan; Department of Laboratory Medicine, Tianjin Haihe Hospital, Tianjin Institute of Respiratory Diseases, Tianjin Medical University, Tianjin, 300350 China; Institute of Toxicology, School of Public Health, Shandong University, Jinan, Shandong 250012 China; Department of Geriatrics, Beijing Tiantan Hospital, Capital Medical University, Beijing, 100050 China; Department of Pathology, Peking University Health Science Center, Beijing, 100083 China

**Keywords:** α-Synuclein, gp91^*phox*^, Superoxide, Neuronal damage, Microglia

## Abstract

**Background:**

Misfolded α-synuclein (α-Syn) aggregates participate in the pathogenesis of synucleinopathies, such as Parkinson’s disease. Whereas much is known about how the various domains within full-length α-Syn (FL-α-Syn) contribute to the formation of α-Syn aggregates and therefore to their neurotoxicity, little is known about whether the individual peptides that can be generated from α-syn, possibly as intermediate metabolites during degradation of misfolded α-Syn aggregates, are neurotoxic themselves.

**Methods:**

A series of synthesized α-Syn peptides, corresponding to the locus in FL-α-Syn containing alanine 30, substitution of which with a proline causes a familial form of Parkinson’s disease, were examined for their capacity of inducing release of microglial superoxide. The neurotoxicity of these peptides was measured according to their influence on the ability of neuroglial cultures deficient in gp91^*phox*^, the catalytic unit of NADPH oxidase (Nox2), or wild-type cultures to take up ^3^H-labeled dopamine and on the number of tyrosine hydroxylase-staining-positive neurons. Western blots and confocal images were utilized to analyze membrane translocation of p47^*phox*^ and p67^*phox*^, phosphorylation of p47^*phox*^ and Erk1/2 kinase, and binding of α-Syn peptides to gp91^*phox*^. Activation of brain microglia in mice injected with α-Syn peptides was demonstrated by immunostaining for major histocompatibility complex (MHC)-II along with qPCR for Iba-1 and MHC-II.

**Results:**

We report α-Syn (29-40) as a specific peptide capable of activating microglial Nox2 to produce superoxide and cause dopaminergic neuronal damage. Administered to mice, this peptide also activated brain microglia to increase expression of MHC-II and Iba-1 and stimulated oxidation reaction. Exploring the underlying mechanisms showed that α-Syn (29-40) peptide triggered Nox2 to generate extracellular superoxide and its metabolite H_2_O_2_ by binding to the catalytic unit gp91^*phox*^ of Nox2; diffusing into cytosol, H_2_O_2_ activated Erk1/2 kinase to phosphorylate p47^*phox*^ and p67^*phox*^ and further activated Nox2, establishing a positive feedback loop to amplify the Nox2-mediated response.

**Conclusions:**

Collectively, our study suggests novel information regarding how α-Syn causes neuronal injury, possibly including mechanisms involving abnormal metabolites of α-Syn aggregates.

## Background

Aggregation of α-synuclein (α-Syn) in the central nervous system (CNS) is a fundamentally important pathological process involved in the development and progression of synucleinopathies including Parkinson’s disease (PD) [1]. α-Syn aggregates, predominantly in oligomeric forms, are thought to be capable of causing neurodegeneration either by directly damaging neurons or by activating microglia [2–5], immune cells in the CNS, to produce inflammatory mediators (e.g., tumor necrosis factor-α (TNF-α)) and reactive oxygen species (ROS) that are neurotoxic [3, 6].

Native α-Syn, composed of 140 amino acids, is traditionally divided into three domains: residues 1–60 form the apolipoprotein-binding domains; residues 61–95 make up a central hydrophobic fragment with a non-amyloid-β component (NAC) region; and residues 96–140 construct a proline-rich region [7]. The physiological function of α-Syn, though it remains to be characterized further, includes the regulation of vesicular transport and neurotransmitter release [8, 9]. In pathological conditions, α-Syn can aggregate, leading to neurodegeneration, and it appears that various regions of α-Syn contribute to the process following different mechanisms of action. Specifically, the NAC region tends to form β-sheets, via a nucleation-dependent kinetic mechanism [10–12]. The apolipoprotein-binding domain also plays a role in aggregation, especially when mutated. For instance, the familial PD-related A30P (alanine 30 to proline-mutated α-synuclein) and A53T (alanine 53 to threonine-mutated α-synuclein) mutations, in which the alanine (A) 30 is replaced by a proline (P) and the alanine (A) 53 by a threonine (T), respectively, dramatically accelerate the initial oligomerization of α-Syn [13–15]. Remarkably, our previous experiments supported the notion that the A30P or A53T forms of the full-length protein (FL-α-Syn) activated microglia more potently than their corresponding wild type [16] and led to greater dopaminergic (DA) neuronal damage. This finding suggests that specific sequences of amino acids in α-Syn could be essential to enhanced neurotoxicity by activating microglia more effectively.

To begin testing this hypothesis, in this investigation, we compared the ability of various peptides corresponding to the A30P region to activate microglia and identified a specific peptide α-Syn (29–40) (i.e., consisting of amino acids A29-V40 of α-Syn) that can directly activate microglial NADPH oxidase (Nox2) to produce superoxide by binding to gp91^*phox*^, the catalytic subunit of this enzyme. The discovery was further validated in vivo, and the underlying mechanisms were found to be related to the phosphorylation of p47^*phox*^ by Erk1/2 kinase, which established a positive feedback loop to achieve a full activation of Nox2.

## Methods

### Experimental animals

Mice lacking in gp91^*phox*^ (gp91^*phox*−/−^) and their wild-type (WT) controls with a C57BL/6J background were provided by the Jackson Laboratory. Animal housing, breeding, and experiments were approved by the Institutional Animal Care Committee of the National Institute of Environmental Health Sciences and performed by strictly following the National Institutes of Health guidelines.

### Animal models

To determine whether the tested α-Syn peptide can pass across the blood-brain barrier (BBB) and diffuse into the interstitial tissues in the brain, mice were injected with saline or fluorescein isothiocyanate (FITC)-labeled α-Syn peptide (5 mg/kg) (ABI Scientific, Inc. Sterling, VA) along with Texas red-labeled lectin (30 μl/mouse) (Vector Laboratories, Inc. Burlingame, CA) for 6 h. The brain tissues were sectioned and stained by using anti-ionized calcium-binding adaptor molecule 1 (Iba-1) antibody (Wako Chemicals USA, Richmond, VA) and 4′,6-diamidino-2-phenylindole, dihydrochloride (DAPI, a fluorescent dye for nuclei) (Sigma-Aldrich, St. Louis, MO). To examine whether injection of α-Syn peptide leads to microglial activation, mice were injected with saline or unlabeled α-Syn peptide (5 mg/kg) for 24 h and further injected with Texas red-labeled lectin 10 min before sacrifice. Brain tissues were harvested and sectioned (30 μm in thickness) for staining nuclei, Iba-1 (using 019-19741 Ab; Wako Chemicals, Richmond, VA), and major histocompatibility complex II (MHC-II) (using M5/114.15.2 Ab; BioLegend, San Diego, CA) or for biochemical assays (e.g., real-time quantitative PCR (RT-qPCR) and malondialdehyde (MDA)). See below for details.

### Preparation of α-Syn aggregates

Dissolved in water at 2 mg/ml, recombinant human α-Syn (rPeptide, Bogart, GA) (endotoxin level: <0.024 EU/μg) was incubated with agitation at 37 °C for 7 days to allow for α-Syn to form aggregates [4, 17]. The concentration of α-Syn aggregates is calculated in terms of the initial concentration of synuclein.

### Design and synthesis of α-Syn peptides

Peptides comprising 12 amino acid residues were chemically synthesized (ABI Scientific, Sterling, VA) according to the amino acid sequence covering the alanine (A) 30-containing loci, which are located within the N-terminal amphipathic region. In the synthesized mutant α-Syn peptides, the alanine (A) 30 was replaced by a P (A30P mutants). The first mutated peptide spans A19 to P30. Shifts occur every two amino acid residues between adjacent peptides. The corresponding amino acid sequences (with the replaced amino acids marked in italics) are AEKTKQGVAEA*P* for A19-P30 (A30P), KTKQGVAEA*P*GK for K21-K32 (A30P), KQGVAEA*P*GKTK for K23-K34 (A30P), GVAEA*P*GKTKEG for G25-G36 (A30P), AEA*P*GKTKEGVL for A27-L38 (A30P), and A*P*GKTKEGVLYV for A29-V40 (A30P). In the screening test using O_2_^• −^ production assay, raw peptides with a purity of approximately 75 % were used individually or together (referred to as “AP pooled”) to stimulate mouse microglia. The experiments were performed following a double blind strategy. Based on the results of screening tests, specific peptides with a purity >98 % were selected to validate the screening test.

### Isolation of microglia

Microglia were prepared as previously described [17]. Briefly, brain cells of mouse pups were collected on postnatal day one (P1) and seeded into poly-d-lysine-coated (Sigma-Aldrich, St. Louis, MO) T175 flasks which were filled with Dulbecco’s modified Eagle medium (DMEM)-F12 (Life Technologies, Grand Island, NY) media containing 10 % fetal bovine serum, 100 U/ml penicillin and streptomycin, 2.0 mM glutamine, 2.0 mM nonessential amino acids, and 1.0 mM sodium pyruvate. Two weeks later, microglia were suspended and harvested by shaking the flasks at 180 rpm for 30 min.

### Primary neuron-glia, neuron-astrocyte, and reconstituted neuron-glia cultures

Neuron-glia cultures were established by following the procedure described previously [18]. The cells of mesencephalic tissues were collected from mouse or rat embryos on embryonic day fourteen (E14) and seeded onto poly-d-lysine-coated 24-well plates in DMEM media (Gibco, Grand Island, NY) supplemented with 10 % fetal bovine serum, 10 % horse serum, 2.0 mM glutamine, 2.0 mM nonessential amino acids, 100 U/ml penicillin and streptomycin, 1.0 mM sodium pyruvate, and 5.6 mM glucose. Typically, immunostaining demonstrated that intact neuron-glia cultures were composed of approximately 40 % neurons (NeuN immunopositive cells), 50 % astrocytes (GFAP immunopositive cells), and 10 % microglia (OX-42 immunopositive cells) [18]. To make neuron-astrocyte cultures, the cells of mesencephalic tissues were collected and seeded into the wells of 24-well plates exactly as in the preparation of neuron-glia cultures. Forty-eight hours after seeding the cells, 2 mM l-leucine methyl ester (Sigma-Aldrich, St. Louis, MO), a compound that is capable of removing microglia but has no influence on the growth of other cell types, was added to each well. Six days after the initial cell seeding, neuron-astrocyte cultures were achieved, which usually contain 44 % neurons, 55 % astrocytes, and less than 0.5 % microglia. To prepare reconstituted neuron-glia cultures, an identical number of freshly isolated WT or gp91^*phox−/−*^ mouse microglia were loaded to rat neuron-astrocyte cultures on day 6 after seeding cells, allowing the newly added microglia to establish cell-cell interactions with neurons and astrocytes. These reconstituted neuron-glia cultures have the same cellular composition as that in the original neuron-glia cultures, including approximately 40 % neurons, 50 % astrocytes, and 10 % microglia. The next day, these reconstituted neuron-glia cultures along with neuron-glia cultures and neuron-astrocyte cultures were treated by adding 150 EU/ml lipopolysaccharide (LPS) (Calbiochem, San Diego, CA) or 1 μM α-Syn peptides. In some experiments, dimethyl sulfoxide (DMSO) or 0.25 mM apocynin (Apo) was added to the cultures 30 min before loading LPS or α-Syn peptides. Seven days after the treatments, all of the cultures were either subjected to dopamine (DA) uptake measurements or immunochemically stained for tyrosine hydroxylase (TH).

### **O**_**2**_^• −^ production assay

**O**_**2**_^• −^ produced by mouse microglia was detected based on the superoxide dismutase (SOD)-inhibitable reduction of the tetrazolium salt WST-1 (Santa Cruz Biotech. Dallas, TX) [19]. Briefly, seeded onto 96-well plates overnight, mouse microglia were washed with Hanks’ balanced salt solution (HBSS) without phenol red followed by adding to each well with 50 μl of HBSS with or without 50 U/ml SOD (Sigma-Aldrich, St. Louis, MO), 50 μl of HBSS containing 1 mM WST-1, and 50 μl of HBSS containing the vehicle control or 1 μM of each α-Syn peptide. The absorbance at 450 nm of each sample was immediately measured using a spectrophotometer (Molecular Devices, Sunnyvale, CA) every 2 min for 30 min. The level of O_2_^• −^ production was calculated in terms of the difference between the absorbance values in the presence and absence of SOD.

### Measurement of DA uptake

After being stimulated with 150 EU/ml LPS, 250 nM α-Syn aggregates, or 1.0 μM α-Syn peptides, neuron-glia, neuron-astrocyte, and reconstituted neuron-glia cultures were incubated for 20 min at 37 °C with ^3^H-labeled DA (PerkinElmer Life Sciences, Santa Clara, CA) in Krebs-Ringer buffer (16 mM sodium phosphate, 119 mM NaCl, 4.7 mM KCl, 1.8 mM CaCl_2_, 1.2 mM MgSO_4_, 1.3 mM EDTA, and 5.6 mM glucose; pH 7.4). Cells were washed with cold Krebs-Ringer buffer and lysed in 1 N NaOH. The radioactivity of the lysates was measured using a liquid scintillation counter (PerkinElmer, Santa Clara, CA) and calculated by subtracting nonspecific DA uptake detected in samples in the presence of 10 μM mazindol (Sigma-Aldrich, St. Louis, MO) from the results of samples that did not include mazindol. Data were presented as the percentage of controls in the same batch of cell cultures [4].

### IC or IF staining

For immunochemical (IC) staining, neuron-glia, neuron-astrocyte, and reconstituted neuron-glia cultures were fixed after stimulation with either 150 EU/ml LPS or 1 μM α-Syn peptides for 7 days. Endogenous peroxidase in the fixed cultures was neutralized with 1 % hydrogen peroxide for 20 min followed by sequentially incubating the cultures together with a TH antibody (Chemicon International, Billerica, MA), biotinylated secondary antibody, and ABC reagents (Dako, Carpinteria, CA). The reaction was revealed by adding 3,3-diaminobenzidine (Thermo Scientific, Waltham, MA) and analyzed using a microscopy coupled with a ×10 objective (Nikon, Tokyo, Japan). The amount of TH-positive cells, which represent dopaminergic neurons, was calculated as the percentage of controls from the same batch of cell cultures [16]. For immunofluorescence (IF) staining of p47^*phox*^ and CD11b, live microglia were first stimulated by 100 nM phorbol 12-myristate 13-acetate (PMA) or 1 μM α-Syn peptides for 30 min at 37 °C. Cells were further incubated on ice with rat anti-mouse CD16/32 IgG (BD Biosciences, San Jose, CA), which blocks Fc receptors on microglia, followed by immunostaining with an APC-conjugated anti-mouse CD11b Ab (BioLegend, San Diego, CA) for 30 min. After being fixed in 4.0 % paraformaldehyde and permeabilized in phosphate-buffered saline (PBS) containing 0.05 % Triton-X, cells were stained for p47^*phox*^ using Alexa 488-labeled anti-mouse antibody (Bioss Inc. Woburn, MA) and were further stained by DAPI to visualize the nuclei. To demonstrate the localization of gp91^*phox*^ and A29-V40 peptide or A19-A30 peptide, freshly isolated WT or gp91^*phox−/−*^ microglia were fixed in 4 % paraformaldehyde and permeabilized by 0.05 % Triton-X. After preincubation with rat anti-mouse CD16/32 IgG, cells were reacted with mouse anti-gp91^*phox*^ Ab (Santa Cruz Biotech. Dallas, TX), followed by adding Alexa 568-labeled secondary Ab. Samples were further incubated with FITC-labeled A29-V40 peptide or FITC-labeled A19-A30 peptide along with DAPI and analyzed with a Zeiss 510 confocal microscopy (Carl Zeiss, Pleasanton, CA). For immunofluorescent staining in thick tissues [20], brain slices (30 μm in thickness) were fixed in 4 % paraformaldehyde for 2 h and blocked in PBS containing 0.5 % Triton-X, 5 % fetal bovine serum, and 5 % goat serum at room temperature (RT) for 2 h. The tissues were further incubated with an anti-Iba-1 Ab (Wako Chemicals, Richmond, VA) in cold room overnight. After multiple washes, samples were incubated with an Alexa 647-conjugated goat anti-rabbit secondary Ab (Life Technologies, Grand Island, NY) along with an Alexa 488-labeled rat anti-mouse MHC-II Ab (M5/114.15.2 Ab; BioLegend, San Diego, CA) and DAPI at RT for 2 h. After washes, tissues were imaged by using a Zeiss 510 confocal microscopy (Carl Zeiss, Pleasanton, CA).

### Detection of membrane translocation of p47^phox^ and p67^phox^ via Western blot

Highly aggressively proliferating immortalized (HAPI) cells were stimulated using 0.1 % BSA, 100 nM PMA, or 1 μM each α-Syn peptide and then lysed in a hypotonic lysis buffer (1 mM Tris, 1 mM KCl, 1 mM EGTA, 1 mM EDTA, and 0.1 mM DTT) supplemented with 10 mg/ml of the protease inhibitor mixture (Thermo Scientific, Waltham, MA) and 1 mM PMSF (Thermo Scientific, Waltham, MA). After dounce homogenization (20–25 St, tight pestle A), the cell lysates were centrifuged at 1600×*g* for 15 min, and the supernatants were further centrifuged at 40,000×*g* for 2 h. The high-speed centrifugation supernatants, which contain the cytosolic fraction, were then harvested. Meanwhile, the plasma membrane fragments were collected by dissolving the pellets in 1 % nonidet P-40 hypotonic lysis buffer. Proteins in both the high-speed supernatants and the re-suspended pellets were separated by running SDS/PAGE gels and were transferred to polyvinylidene difluoride (PVDF) membranes, followed by immune-blotting using anti-p47^*phox*^, anti-p67^*phox*^, and anti-gp91^*phox*^ Abs (Cell Signaling, Danvers, MA). The relative amount of each protein was quantified using the Quantity One program (Bio-Rad, Redmond, WA). The glyceraldehyde-3-phosphate dehydrogenase (GAPDH) in whole-cell lysates were immunoblotted by an Ab (Cell Signaling, Danvers, MA) as a loading control [21].

### Co-immunoprecipitation

Flag-fused α-Syn peptides (ABI Scientific Inc., Sterling, VA) and cell lysates, which were collected from WT or gp91^*phox−/−*^ mixed glial cultures or enriched microglia by using IP lysis buffer (Thermo Scientific, Waltham, MA), were mixed in a cold room overnight in the presence of protease inhibitor cocktail (Thermo Scientific, Waltham, MA). Simultaneously, goat anti-gp91^*phox*^ polyclonal antibodies (Santa Cruz Biotech., Dallas, TX) or control IgGs were conjugated to protein G magnetic beads using a Dynabeads protein G immunoprecipitation kit (Life Technologies, Grand Island, NY) at RT for 10 min. Next, the α-Syn peptides and cell lysate mixtures were incubated with the antibody- or control IgG-conjugated protein G magnetic beads at RT for 30 min. After washing, the beads were boiled in Laemmli sample buffer and the supernatants were resolved using SDS-PAGE. The proteins were transferred to Nitrocellulose membranes (0.2 μm, Bio-Rad Laboratories Inc., Redmond, WA) and immunoblotted using mouse antibodies against Flag (Origene, Rockville, MD), gp91^*phox*^ (Santa Cruz Biotech. Dallas, TX), or tubulin (Abcam, Cambridge, MA).

### Isolation of total RNA and qPCR

Isolation of total RNA from the brain tissues was performed by using TRIzol according to the manufacturer’s instructions (Life Technologies, Grand Island, NY). Quality and quantity of total RNA were measured by using the Agilent 2100 Bioanalyzer (Agilent Technologies, Inc., Santa Clara, CA). Reverse transcriptase reactions were run on 1 μg of total messenger RNA (mRNA) by using the reverse transcription system (Promega, Madison, WI). Quantitative PCR was performed by using a Light Cycler 480 (Roche, Indianapolis, IN) and the SYBR Green I Master (Roche, Indianapolis, IN) according to the manufacturer’s instructions. Primers for mouse Iba-1 were 5′-GGATTTGCAGGGAGGAAAAG-3′ and 5′-TGGGATCATCGAGGAATTG-3′; primers for mouse MHC-II were 5′-CCG TCA CAG GAG TCA GAA AGG-3′ and 5′-CGG AGC AGA GAC ATT CAG GTC-3′; and primers for β-actin were 5′-CCCAAGGCCAACCGCGAGAAGAT-3′ and 5′-GTCCCGGCCAGCCAGGTCCAG-3′.

### MDA assays

Mouse brain tissues were homogenized on ice using 300 μl of the MDA lysis buffer according to the manufacturer’s guidance (Sigma-Aldrich, St. Louis, MO) (with 3 μl of butylated hydroxytoluene [100X]), followed by centrifugation (13,000×*g*, 10 min) to remove insoluble materials. The supernatant (200 μl) from each homogenized sample was placed into a micro-centrifuge tube and incubated with 600 μl of thiobarbituric acid solution at 95 °C for 60 min. Cooled to RT in an ice bath for 10 min, 200 μl of mixtures was pipetted into a 96-well microplate and analyzed by using a fluorescence plate reader (Ex: 532 nm; Em: 553 nm) (Molecular Devices, Sunnyvale, CA).

### Statistical analysis

Presented as the means ± SEM, data were evaluated using analysis of variance (ANOVA) followed by multiple comparison tests. If the *P* value is less than 0.05, results are considered statistically significant.

## Results

### A29-V40 (A30P) α-Syn peptide stimulates microglia to produce superoxide (**O**_**2**_^• −^)

**Fig. 1:**
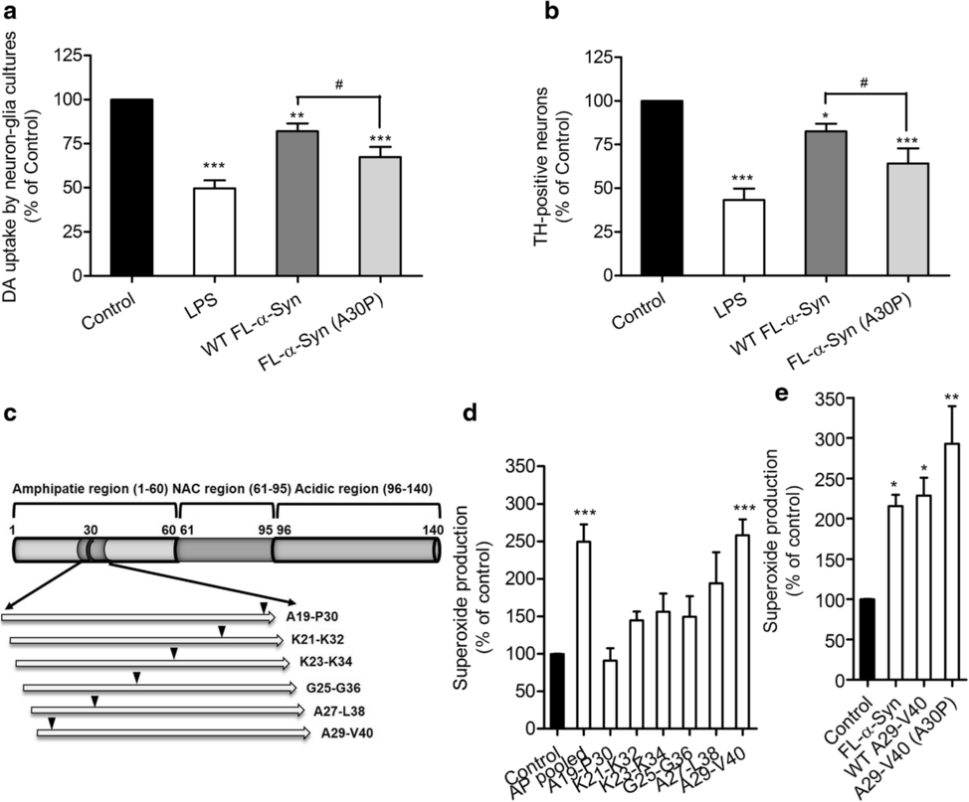
Synthesis of α-Syn peptides and production of microglial superoxide (**O**_**2**_^• −^) upon stimulation of full-length α-Syn (FL-α-Syn) or its peptides. **a** Changes in the ability of rat neuron-glia cultures to take up ^3^H-labeled DA after exposure to WT FL-α-Syn aggregates, their corresponding mutant A30P, or LPS which serves as an experimental control. **b** Changes in the number of TH-positive neurons in rat neuron-glia cultures after exposure to WT FL-α-Syn aggregates, their corresponding mutant A30P, or LPS. **c** A schematic diagram illustrating the structure of full-length α-Syn (FL-α-Syn) and the strategy for α-Syn peptide synthesis. The location of the A30P point mutation is marked by an *arrow*. **d** Levels of extracellular superoxide in mouse microglia-enriched cultures upon the stimulation of BSA (control) or raw α-Syn peptides in which each peptide has a purity of approximately 75 % and harbors the point mutation A30P. **e** Based on the results collected from the screening test in **b**, highly purified AP71 peptide, i.e., A29-V40 (A30P), and its corresponding WT, namely A29-V40 (purity >98 %), were selected to further stimulate mouse microglia. Production of extracellular superoxide was measured. BSA treatment serves as a basal control; in contrast, FL-α-Syn aggregates act as a positive control. In **d**, **e**, ANOVA analysis plus Newman-Keuls multiple comparisons were performed. *P < 0.05, **P < 0.01, and ***P < 0.001, compared to the BSA basal controls

Aggregation of FL-α-Syn can be facilitated by the presence of A30P mutation in its apolipoprotein-binding domain, therefore leading to greater neurotoxicity by directly affecting neurons or by activating microglia [16, 22]. To determine the effects of A30P mutation on the ability of α-Syn to induce dopaminergic neuronal damage via microglia activation, we first compared the effects of WT or A30P FL-α-Syn on DA uptake (which has been shown to correlate with the function and structural integrity of dopaminergic neurons [4, 23–25]) in neuron-glia cultures, which consist of approximately 40 % neurons, 50 % astrocytes, and 10 % microglia [18]. In this study, LPS was used as a positive control because adding 150 EU/ml LPS to neuron-glia cultures consistently causes an approximately 50 % loss of dopaminergic neurons. We found that, as demonstrated previously [16], FL-α-Syn aggregates featuring the A30P substitution are more toxic to dopaminergic neurons than WT, leading to an even lower ability of neurons to take up dopamine (DA), and resulting in fewer surviving TH-positive cells, which represent dopaminergic neurons in the cultures (Fig. 1a, b).

These findings suggest that the regions covering the A30P locus possibly contribute to activation of microglia and the ensuing neuroinflammation-mediated dopaminergic neuronal damage. This hypothesis led us to systematically examine the capacity of peptides corresponding to the amino acid sequences in the proximity of alanine 30 to activate microglia and induce neuronal damage. For this purpose, we synthesized six peptides consisting of 12 amino acids, all of which included the A30P substitution, following the strategy illustrated in Fig. 1c. For each consecutive peptide within this set, the first included amino acid was shifted by two amino acids toward the C-terminus. These peptides with a purity of 75 %, either individually or together, were added to stimulate primary mouse microglia. Superoxide released from microglia was significantly increased (149.4 ± 23.2 % increase) after exposure to the mixture of six peptides harboring the A30P mutation. However, when each of these peptides was added individually to mouse microglial cultures, A29-V40 (A30P, A**P**GKTKEGVLYV) was the only peptide capable of stimulating the release of microglial superoxide (Fig. 1d) approximately 158.0 ± 21.4 % more than the BSA control. In another set of experiments, we tested whether the A30P substitution was critical for generating the elevated microglial response using highly purified peptides (purity >98 %) and found that WT and A30P-mutated A29-V40 peptides were equipotent in activating microglia to release superoxide (128.6 ± 22.2 % and 192.6 ± 47.1 % increase, respectively, compared to the BSA control), suggesting that at the concentration of 1.0 μM, the presence of A30P mutation does not enhance peptide-induced microglial release of superoxide (Fig. 1e). This is different from our previous finding that the increase of superoxide production induced by A30P-mutated FL-α-Syn aggregates (≈250 %) was significantly greater than by its WT form (≈90 %) [16].

### Unlike FL-α-Syn aggregates, WT A29-V40 peptide exhibits similar toxicity to dopaminergic neurons as its A30P mutant

**Fig. 2:**
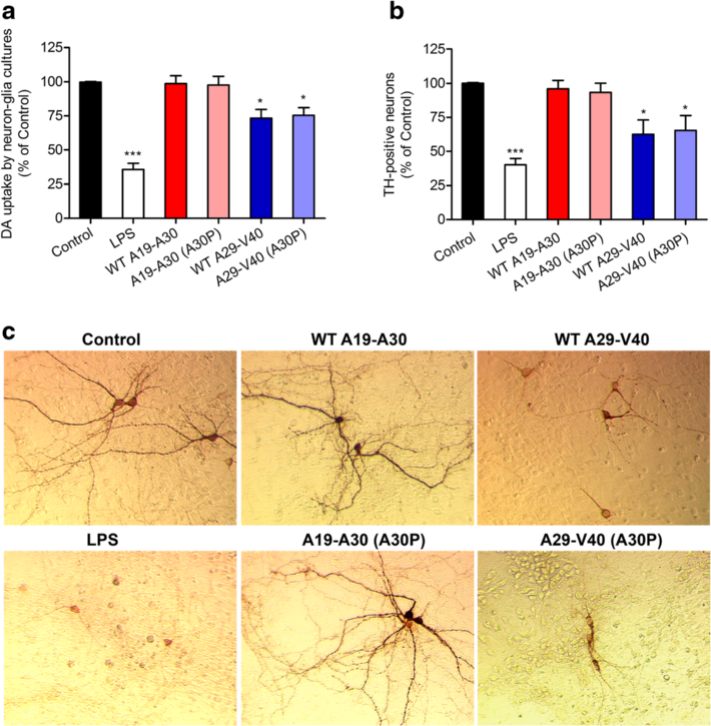
Changes in the function and viability of dopaminergic neurons after exposure to WT FL-α-Syn or A29-V40 peptide, the corresponding A30P mutants, or LPS. **a** Changes in the ability of rat neuron-glia cultures to take up ^3^H-labeled DA after exposure to WT α-Syn peptides A19-A30 or A29-V40, their corresponding mutants A19-A30 (A30P) or A29-V40 (A30P), or LPS. **b** Changes in the number of TH-positive neurons in rat neuron-glia cultures after exposure to WT α-Syn peptides A19-A30 or A29-V40, their corresponding mutants A19-A30 (A30P) or A29-V40 (A30P), or LPS. *n* = 5–6; *P < 0.05, **P < 0.01, and ***P < 0.001, compared with the basal controls; ^#^ *P* < 0.05, compared as indicated. **c** Representative images, which were captured by a ×10 objective, showing TH-positive neurons in rat neuron-glia cultures treated with various stimuli

Given the finding that A30P-mutated FL-α-Syn induced greater dopaminergic neuronal damage than WT, but WT A29-V40 peptide and its corresponding A30P-mutant activate microglia equally, we compared the effects of A29-V40 peptides with or without A30P on neurons in neuron-glia cultures. Exposure of cultures to either WT or mutated A29-V40 (A30P) peptide led to a reduction in DA uptake (Fig. 2a) and significantly decreased the number of surviving TH-positive cells to a similar level (Fig. 2b). However, unlike what occurred with FL-α-Syn aggregates, the presence of the point mutation A30P in A29-V40 peptide failed to cause additional damage of dopaminergic neurons (Fig. 1a, b and Fig. 2a–c), suggesting that different mechanisms might, respectively, be involved in FL-α-Syn aggregate- and A29-V40 peptide-induced neuronal toxicity. In contrast to A29-V40 peptides, neither WT A19-A30 control peptide nor its A30P mutant, which was not able to induce the release of microglial superoxide (Fig. 1d), impaired the function and viability of dopaminergic neurons (Fig. 2a, b). Moreover, morphological analyses showed that the neurites of surviving neurons were shorter in the cultures treated by A29-V40 peptides than in the vehicle controls. In contrast, the peptide A19-A30, either WT or A30P mutant, did not cause significant changes in neuronal morphology (Fig. 2c). Together, our data suggest that the A29-V40 peptide is a specific α-Syn fragment that is independently capable of inducing dopaminergic neuronal damage and that ROS generation is critical in the process.

### Nox2 activity is essential to A29-V40 peptide-induced damage of dopaminergic neurons

**Fig. 3:**
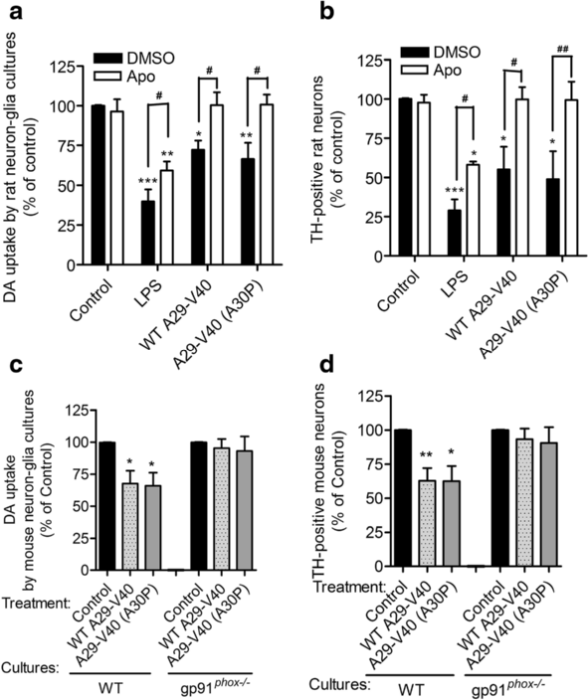
Effect of inhibition or deficiency of Nox2 activity on the function and viability of dopaminergic neurons after exposure to WT or mutated A29-V40 peptides or LPS. **a** Changes in the ability of rat neuron-glia cultures to take up ^3^H-labeled DA after exposure to WT α-Syn peptide A29-V40, its corresponding mutant A29-V40 (A30P), or LPS in the presence or absence of the Nox2 inhibitor apocynin (Apo). **b** Changes in the number of TH-positive neurons in rat neuron-glia cultures after exposure to WT α-Syn peptide A29-V40, its corresponding mutant A29-V40 (A30P), or LPS in the presence or absence of Apo. **c** Changes in the ability of WT or gp91^*phox−/−*^ mouse neuron-glia cultures to take up ^3^H-labeled DA after exposure to WT α-Syn peptide A29-V40 and its corresponding mutant A29-V40 (A30P). **d** Changes in the number of TH-positive neurons in WT or gp91^*phox−/−*^ mouse neuron-glia cultures after exposure to WT α-Syn peptide A29-V40 and its corresponding mutant A29-V40 (A30P). In **a**, **b**, two-way ANOVA and Bonferroni’s post hoc test were performed. *n* = 5; *P < 0.05, **P < 0.01, ***P < 0.001, compared with the corresponding DMSO-treated controls; ^#^ *P* < 0.05 and ^##^ *P* < 0.01, compared as indicated. In **c**, **d**, one-way ANOVA and Dunnett’s test were performed. *n* = 5; *P < 0.05, **P < 0.01, compared with the corresponding basal controls

Because Nox2 is a key enzyme regulating the release of superoxide from microglia, we examined whether this enzyme contributes to A29-V40-induced dopaminergic neuronal damage. To this end, neuronal loss was analyzed in neuron-glia cultures which were pretreated with the vehicle control DMSO or Apo, a Nox2 inhibitor, followed by adding A29-V40 peptide, either WT or mutated. Consistent with the data shown in Fig. 2, the presence of A29-V40 peptides markedly decreased the value of DA uptake and the number of surviving dopaminergic neurons, compared to that of the controls (Fig. 3a, b). Pretreatment with Apo fully restored the ability to take up DA and the number of surviving dopaminergic neurons in the cultures, which were stimulated by A29-V40 peptides. However, this compound could only partially restore the level of DA uptake and the number of surviving dopaminergic neurons in LPS-treated cultures (Fig. 3a, b), indicating that in addition to Nox2, other mechanisms may also contribute to the LPS-induced damage of dopaminergic neurons. While comparing the responses of WT neuron-glia cultures and those deficient in gp91^*phox*^ (gp91^*phox−/−*^), the catalytic unit of Nox2, to either WT or mutated A29-V40 peptides, we found that A29-V40 peptides failed to impair the ability to take up DA and cause the loss of dopaminergic neurons in the cultures deficient in gp91^*phox*^ (Fig. 3c, d). Together, our data suggested that the activity of Nox2 is required to mediate peptide A29-V40-induced dopaminergic neuronal damage.

### Nox2 activation in microglia rather than in neuron-astrocytes results in A29-V40 peptide-induced neurotoxicity

**Fig. 4:**
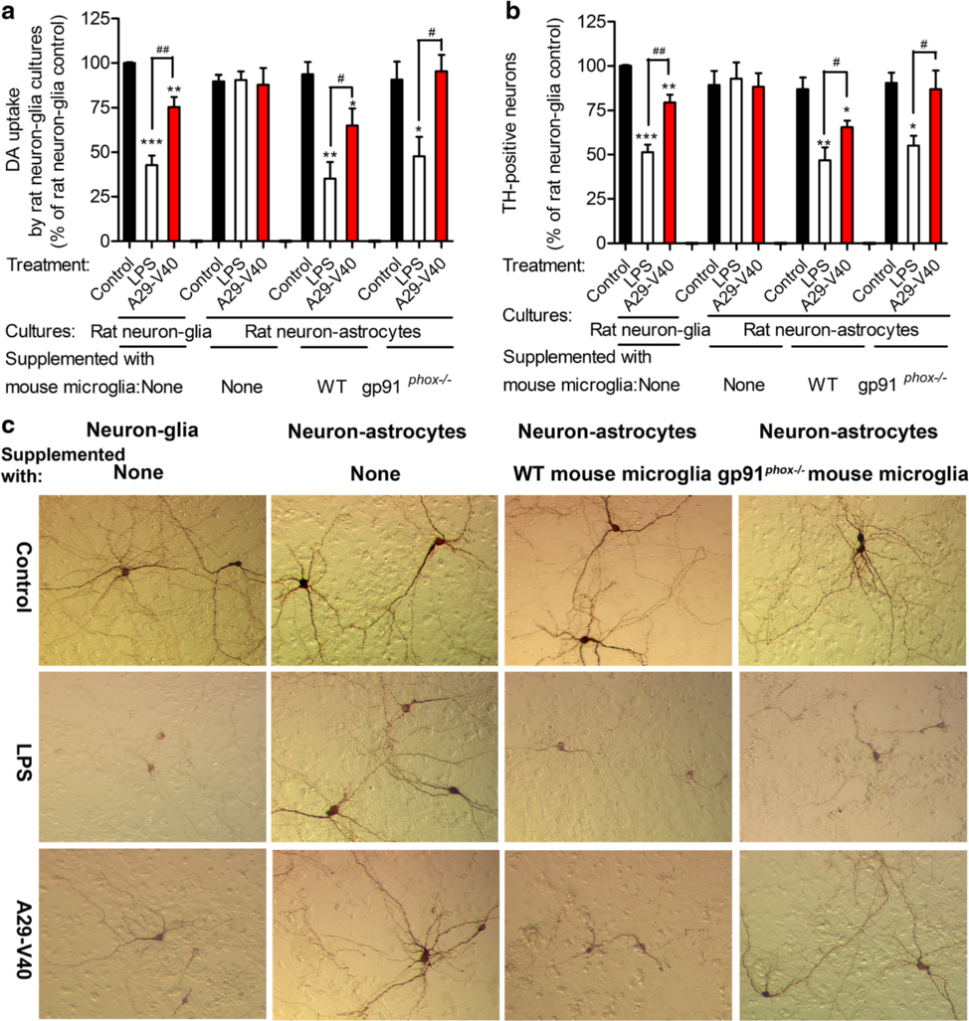
Activation of Nox2 in microglia but not in neurons or astrocytes accounts for A29-V40 peptide-induced loss of the viability and function of dopaminergic neurons. **a** Changes in the ability of rat neuron-glia, neuron-astrocyte cultures, and neuron-astrocyte cultures which were supplemented with WT or gp91^*phox−/−*^ mouse microglia, to take up ^3^H-labeled DA after exposure to WT α-Syn peptide A29-V40 or LPS. **b** Changes in the number of TH-positive neurons in rat neuron-glia, neuron-astrocyte cultures, and neuron-astrocyte cultures which were supplied with WT or gp91^*phox−/−*^ mouse microglia (i.e., reconstituted neuron-glia cultures), after exposure to WT α-Syn peptide A29-V40 or LPS. **c** Representative images showing the morphology of TH-positive neurons in cultures after exposure to WT α-Syn peptide A29-V40 or LPS. One-way ANOVA analyses and Newman-Keuls multiple comparison tests were performed. *n* = 5; *P < 0.05, **P < 0.01, ***P < 0.001 compared with the corresponding controls; ^#^ *P* < 0.05 and ^##^ *P* < 0.01 compared as indicated

Next, we determined the source of Nox2 that participated in peptide A29-V40-induced dopaminergic neuronal damage. Since superoxide production by microglia was used as an index while screening the active α-Syn peptides, we hypothesized that the Nox2 activated by peptides A29-V40 originates in microglia but not in neuron and/or astrocytes. To test this hypothesis, we examined DA uptake by neurons and counted surviving dopaminergic neurons in multiple types of cultures, including rat neuron-glia cultures (containing neurons, astrocytes, and microglia), neuron-astrocyte cultures (containing neurons and astrocytes), and reconstituted neuron-glia cultures (established by adding either WT or gp91^*phox−/−*^ mouse microglia back to rat neuron-astrocyte cultures). Because the A30P substitution did not influence the neurotoxicity of the A29-V40 peptide, we only tested its WT form. As shown in Fig. 4a, whereas peptide A29-V40 reduced DA uptake in WT neuron-glia cultures (the left group of bars), it did not impair DA uptake in neuron-astrocyte cultures that lacked microglia (the left-middle group of bars), meaning that peptide A29-V40 has no direct toxicity to neuron-astrocyte cultures or is incapable of stimulating them to generate toxic products (e.g., O_2_^·–^) to damage themselves. However, once WT microglia had been added back to neuron-astrocyte cultures, peptide A29-V40 re-gained the capacity to damage dopaminergic neurons (the right-middle group of bars). In contrast, adding gp91^*phox−/−*^ microglia to neuron-astrocyte cultures failed to restore the ability of the A29-V40 peptide to induce neuronal damage (the right group of bars). A similar response pattern was observed by counting the number of surviving dopaminergic neurons in these cultures that were exposed to the peptide A29-V40 (Fig. 4b). Moreover, TH-positive neurons cultured with microglia, either pre-existing or added back after purification, had shorter and slimmer processes after the addition of A29-V40 peptide. This response could not be re-created in neuron-astrocyte cultures or neuron-astrocyte cultures supplemented with gp91^*phox−/−*^ microglia (Fig. 4c). LPS treatment impaired the function and viability of dopaminergic neurons to a greater extent than A29-V40 peptide in the presence of microglia, but addition of gp91^*phox*^ knockout microglia only partially restored the loss of neuronal function and viability, suggesting that LPS-induced dopaminergic neuronal damage in our neuron-glia cultures is partially caused by inflammatory mediators (e.g., TNF-α) [26] other than Nox2-derived superoxide (Fig. 4a–c). Although one could argue that some of the observations might be confounded by the fact that mixed species (rat neurons and astrocytes mixed with mouse microglia) were used in the study, it is unlikely that microglia originally from different species cause substantial differences in the neurotoxicity of LPS or peptides, because reconstituted neuron-glia cultures, which were established by loading WT mouse microglia to rat neuron-astrocyte cultures, responded to the stimulation of LPS or A29-V40 peptide very similar to rat neuron-glia cultures (Fig. 4a, b). Moreover, our previous studies showed that superoxide generation by mouse and rat microglia are quite similar. Therefore, our data indicate that the Nox2 activity of microglia accounts for the neurotoxicity caused by the A29-V40 peptide.

### A29-V40 activates Nox2 by binding to gp91^*phox*^, which is located on cell membranes

**Fig. 5:**
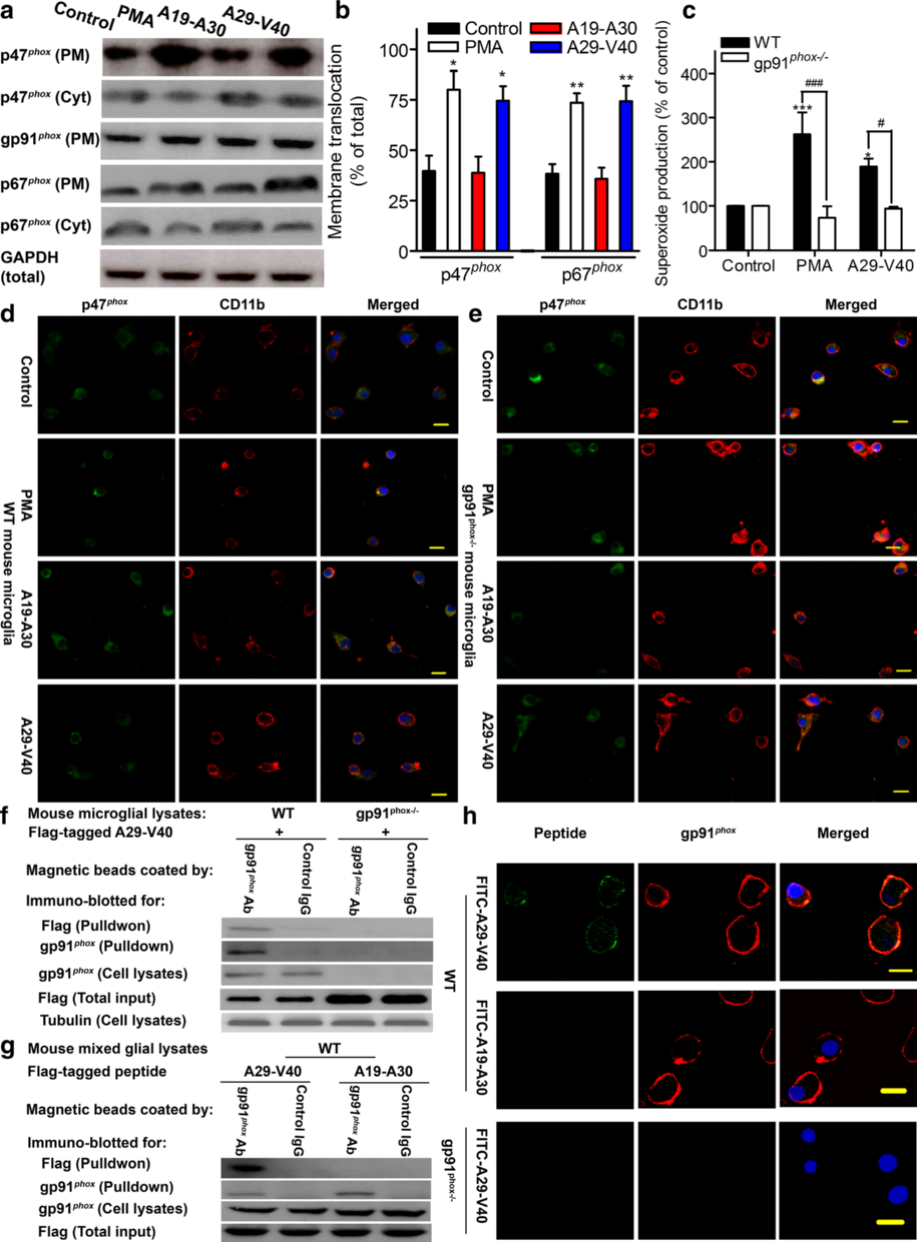
A29-V40 peptide activates Nox2 to produce superoxide via binding to gp91^*phox*^ *.* **a** Membrane translocation of p47^*phox*^ and p67^*phox*^ in rat microglia-derived HAPI cells. After stimulation by PMA or α-Syn peptides A19-A30 and A29-V40, cells were lysed in hypotonic lysis buffer. The membrane fraction and the cytosol were extracted from the lysates and were dissolved in SDS-PAGE and transferred to PVDF membranes. Samples were immunoblotted for p47^*phox*^, p67^*phox*^, gp91^*phox*^, and tubulin. *PM* plasma membrane; *Cyt* cytosol. **b** Quantitative analyses of p47^*phox*^ and p67^*phox*^ membrane translocation according to the immunoblot data as shown in **a**. ANOVA was performed; *n* = 3–5; and *P < 0.05, **P < 0.01 compared to the corresponding basal controls. **c** Superoxide production in mouse microglial cultures. WT or gp91^*phox−*/*−*^ microglia were seeded in 96-well plates. Upon stimulation using PMA (positive control) or α-Syn peptides A29-V40, superoxide production was measured. *n* = 4–6; *P < 0.05 and ***P < 0.001, compared to the corresponding basal control; ^###^ *P* < 0.001, compared as indicated. **d** Representative images showing membrane translocation of WT mouse microglial p47^*phox*^. After stimulation using PMA or α-Syn peptides A19-A30 and A29-V40, the cells were fixed, permeabilized, and immunostained for CD11b and p47^*phox*^. **e** Representative images showing membrane translocation of gp91^*phox*^ null microglial p47^*phox*^. After stimulation using PMA or α-Syn peptides A19-A30 and A29-V40, gp91^*phox*^ null microglia were fixed, permeabilized, and immunostained for p47^*phox*^. **f** Binding of A29-V40 peptide to gp91^*phox*^. Flag-fused A29-V40 peptide was incubated with WT or gp91^*phox−/−*^ microglial lysates, and the mixtures were further incubated with protein G-conjugated magnetic beads which were coated with control IgG or anti-gp91^*phox*^ polyclonal Ab. After washes, beads were boiled and the supernatants were dissolved in SDS-PAGE. Samples were immunoblotted for Flag, gp91^*phox*^, or tubulin. **g** Binding of A29-V40 peptide or A19-A30 peptide to gp91^*phox*^. **h** Representative images illustrating the cell surface binding of FITC-labeled A29-V40 peptide but not A19-A30 peptide to gp91^*phox*^. WT or gp91^*phox−/−*^ microglia were incubated with FITC-labeled A29-V40 peptide or A19-A30 peptide on ice for 30 min and imaged by a confocal microscope. *Bar* = 10 μm

Next, we explored direct evidence that A29-V40 activates microglial Nox2. Activation of Nox2 is mediated by the recruitment of cytosolic phosphorylated p47^*phox*^ and p67^*phox*^ and activated small GTPase Rac to cell membranes [27]. At the membrane, these subunits, along with gp91^*phox*^ and p22^*phox*^ which are normally located on cell membranes, assemble to form an activated multicomponent enzyme complex—Nox2. Therefore, membrane translocation of p47^*phox*^ and p67^*phox*^ usually indicates up-regulation of Nox2 activity. Upon stimulation by either PMA, a well-known protein kinase C (PKC) activator, or A29-V40 peptide, both p47^*phox*^ and p67^*phox*^ were enriched in the extracts of cellular membranes collected from rat microglia-derived HAPI cells. This response occurred with a simultaneous reduction of p47^*phox*^ and p67^*phox*^ in the cytosol. In contrast, A19-A30 peptide failed to increase p47^*phox*^ and p67^*phox*^ membrane translocation (Fig. 5a, b). The ability of A29-V40 peptide to activate microglial Nox2 was further confirmed by comparing the amount of superoxide produced by A29-V40 peptide-stimulated WT and gp91^*phox−/−*^ microglia. Like PMA, A29-V40 peptide markedly accelerated release of superoxide from WT microglia, but not from gp91^*phox−/−*^ lacking the Nox2 catalytic unit (Fig. 5c). Confocal imaging also revealed PMA- and A29-V40 peptide-induced accumulation of p47^*phox*^ on WT mouse microglial membranes, co-localized with microglial cell surface CD11b. However, p47^*phox*^ remained concentrated in the cytosol in A19-A30 peptide-treated cells (Fig. 5d). Intriguingly, none of these stimuli could induce p47^*phox*^ membrane translocation in gp91^*phox*^ null microglia (Fig. 5e), suggesting that regardless of the type of stimuli, gp91^*phox*^ is essential for recruiting and anchoring p47^*phox*^ onto cell membranes.

As a membrane-permeable compound, PMA can enter the cytosol and directly activate PKC to phosphorylate p47^*phox*^ and p67^*phox*^, eventually leading to Nox2 activation via an “inside to outside” signal pathway [27]. However, A29-V40 peptide and PMA share no similarity in biochemical characteristics, prompting us to speculate that PMA and A29-V40 peptide activate Nox2 through distinct pathways. In this regard, previous studies have reported that some small molecules [28, 29] can directly target and activate the Nox2 catalytic unit gp91^*phox*^. Therefore, we tested whether A29-V40 peptide can bind to gp91^*phox*^. Supporting this idea, when gp91^*phox*^ antibody-conjugated magnetic beads were incubated with a mixture of Flag-tagged A29-V40 peptide and WT microglial lysates, both A29-V40 peptide and gp91^*phox*^ were precipitated, while neither was precipitated by beads conjugated to control IgG. However, the beads failed to pull down A29-V40 peptide from the mixture composed of Flag-tagged A29-V40 peptide and gp91^*phox−/−*^ cell lysates (Fig. 5f) or precipitate A19-A30 control peptide from the mixture composed of Flag-tagged A19-A30 peptide and WT cell lysates (Fig. 5g), suggesting that A29-V40 peptide is a specific peptide capable of binding to gp91^*phox*^. This idea was further confirmed by the observation that FITC-labeled A29-V40 peptide could stick to the cell surface of WT but not gp91^*phox−/−*^ microglia whereas A19-A30 peptide failed to bind to the cell surface of WT microglia (Fig. 5h).

### Exposure to A29-V40 peptide activates Erk1/2 to phosphorylate p47^*phox*^ and to amplify Nox2 activation, dependent on the presence of H_2_O_2_

**Fig. 6:**
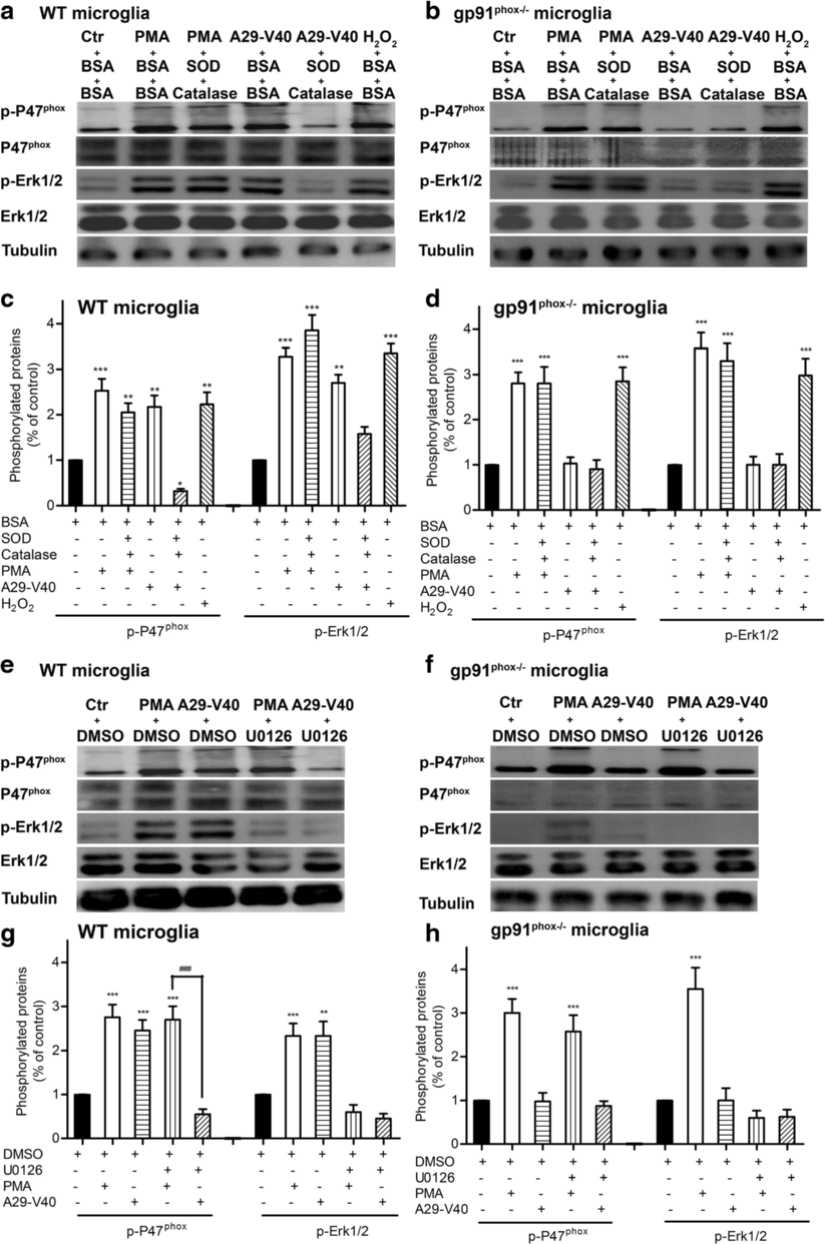
Exposure to A29-V40 peptide activates Erk1/2 to phosphorylate p47phox and to amplify Nox2 activation, dependent on the presence of H_2_O_2_. **a** Effect of the presence of SOD and catalase on the phosphorylation of WT microglial p47^phox^ and Erk1/2. WT mouse microglia were seeded on six-well plates and pretreated with or without SOD and catalase. After stimulation by PMA, α-Syn peptide, or H_2_O_2_ in the presence or absence of SOD and catalase, cells were lysed in IP buffer containing protease inhibitors. Cell lysates were subject to SDS-PAGE and were immunoblotted for p47^*phox*^, phosphorylated p47^*phox*^, Erk1/2, phosphorylated Erk1/2, or tubulin. **b** Effect of the presence of SOD and catalase on the phosphorylation of p47^*phox*^ and Erk1/2 in gp91^*phox*^ null microglia. Microglia deficient in gp91^*phox*^ were seeded on six-well plates and pretreated with or without SOD and catalase. After stimulation by PMA, α-Syn peptide, or H_2_O_2_ in the presence or absence of SOD and catalase, cells were lysed in IP buffer containing protease inhibitors. Cell lysates were subject to SDS-PAGE and were immunoblotted for p47^*phox*^, phosphorylated p47^*phox*^, Erk1/2, phosphorylated Erk1/2, or tubulin. **c** Quantitative analysis of the phosphorylation of p47^*phox*^ and Erk1/2 in WT microglia based on the immunoblot data shown in **a. d** Quantitative analysis of the phosphorylation of p47^*phox*^ and Erk1/2 in gp91^*phox*^ null microglia based on the immunoblot data shown in **b. e** Effect of the presence of Erk inhibitor U0126 on the phosphorylation of WT microglial p47^*phox*^ and Erk1/2. WT mouse microglia were seeded on six-well plates and pretreated with or without U0126. After stimulation by PMA or α-Syn peptide in the presence or absence of U0126, cells were lysed in IP buffer containing protease inhibitors. Cell lysates were subject to SDS-PAGE and were immunoblotted for p47^phox^, phosphorylated p47^phox^, Erk1/2, phosphorylated Erk1/2, or tubulin. **f** Effect of the presence of Erk inhibitor U0126 on the phosphorylation of p47^phox^ and Erk1/2 in gp91 null microglia. Microglia deficient in gp91^*phox*^ were seeded on six-well plates and pretreated with or without U0126. After stimulation by PMA or α-Syn peptide in the presence or absence of U0126, cells were lysed in IP buffer containing protease inhibitors. Cell lysates were subject to SDS-PAGE and were immunoblotted for p47^*phox*^, phosphorylated p47^*phox*^, Erk1/2, phosphorylated Erk1/2, or tubulin. **g** Quantitative analysis of the phosphorylation of WT microglial p47^*phox*^ and Erk1/2 based on the immunoblot data shown in **e. h** Quantitative analysis of the phosphorylation of p47^*phox*^ and Erk1/2 in gp91^*phox*^ null microglia based on the immunoblot data shown in **f**. In **c**, **d**, **g**, and **h**, ANOVA analyses followed by Newman-Keuls multiple comparison tests were performed. *n* = 3–4; *P < 0.05, **P < 0.01, and ***P < 0.001, compared with the corresponding controls. ^###^ *P* < 0.001, compared as indicated

A major result of Nox2 activation is increased extracellular levels of O_2_^• −^, which is spontaneously converted to H_2_O_2_, a reaction that is facilitated by SOD [30]. Since, unlike PMA, A29-V40-induced activation of Nox2 occurs via binding to gp91^*phox*^, we hypothesized that H_2_O_2_ may be the membrane-permeable molecule that transmits the signal to the cytosol. To further investigate this hypothesis, we added SOD and catalase, an enzyme with the ability to break down H_2_O_2_ to H_2_O and O_2_, to mouse microglial cultures before stimulating cells with PMA (an experimental control), A29-V40 peptide, or H_2_O_2_ (a positive control) and examined the effects on downstream mediators. A29-V40 peptide markedly increased the level of both phosphorylated p47^*phox*^ and Erk1/2 in WT microglia while the presence of SOD and catalase attenuated this response. Pretreating WT microglia with SOD and catalase did not affect PMA-induced phosphorylation of p47^*phox*^ and Erk1/2 (Fig. 6a, c), indicating, as expected, that H_2_O_2_ does not mediate this response. In gp91^*phox−/−*^ microglia, A29-V40 peptide was not able to induce phosphorylation of p47^*phox*^ and Erk1/2 in the presence or absence of SOD and catalase. In contrast, p47^*phox*^ and Erk1/2 phosphorylation was induced by direct exposure of gp91^*phox−/−*^ microglia to H_2_O_2_. Therefore, both the presence of gp91^*phox*^ and generation of H_2_O_2_ are necessary for the A29-V40-induced activation of Nox2. Despite the deficiency of gp91^*phox*^ in microglia, PMA retained the ability to induce Erk1/2 and p47^*phox*^ phosphorylation in the presence or absence of SOD and catalase (Fig. 6b, d), further confirming that different mechanisms underlie A29-V40 peptide- and PMA-induced microglial Nox2 activation.

As key components in the Ras-Raf-MEK-ERK signal transduction pathway, ERK1 and ERK2 are related to protein-serine/threonine kinases that may cause phosphorylation of p47^*phox*^ [31]. Therefore, we sought to determine whether Erk1/2 phosphorylation is necessary for p47^*phox*^ phosphorylation. Pretreatment with the Erk1/2 inhibitor U0126 [32] abolished Erk1/2 phosphorylation in PMA or A29-V40 peptide-stimulated WT microglia to levels even lower than controls. This inhibitor also led to a lower level of p47^*phox*^ phosphorylation in A29-V40 peptide-stimulated WT microglia but had no effect on PMA-induced p47^*phox*^ phosphorylation (Fig. 6e, g). Consistent with data shown in Fig. 6b, d, exposure of gp91^*phox−/−*^ microglia to A29-V40 peptide could neither activate Erk1/2 nor induce p47^*phox*^ phosphorylation while stimulation by PMA led to phosphorylation of both Erk1/2 and p47^*phox*^. This suggests that Erk1/2 activation is upstream of p47^*phox*^ phosphorylation in A29-V40 peptide-initiated response, but PMA activates Erk1/2 and p47^*phox*^ independent of gp91^*phox*^. Using U0126 to inhibit Erk1/2 activity did not cause any additive suppression of p47^*phox*^ phosphorylation in A29-V40 peptide-treated gp91^*phox−/−*^ microglia, compared to the DMSO control. This observation suggests that binding of A29-V40 peptide and gp91^*phox*^ is the initial event that activates Nox2 to produce superoxide and hence H_2_O_2_; H_2_O_2_ then enters cells and induces activation of Erk1/2, upstream of p47^*phox*^ phosphorylation, which may amplify A29-V40 peptide-induced Nox2 activation. Despite the suppression of Erk1/2 activity in U0126-pretreated gp91^*phox−/−*^ microglia, PMA still induced p47^*phox*^ phosphorylation (Fig. 6f, h), indicating that this compound activates Nox2 in an Erk1/2-independent manner.

### A29-V40 peptide activates microglia and elicits redox reactions in vivo

**Fig. 7:**
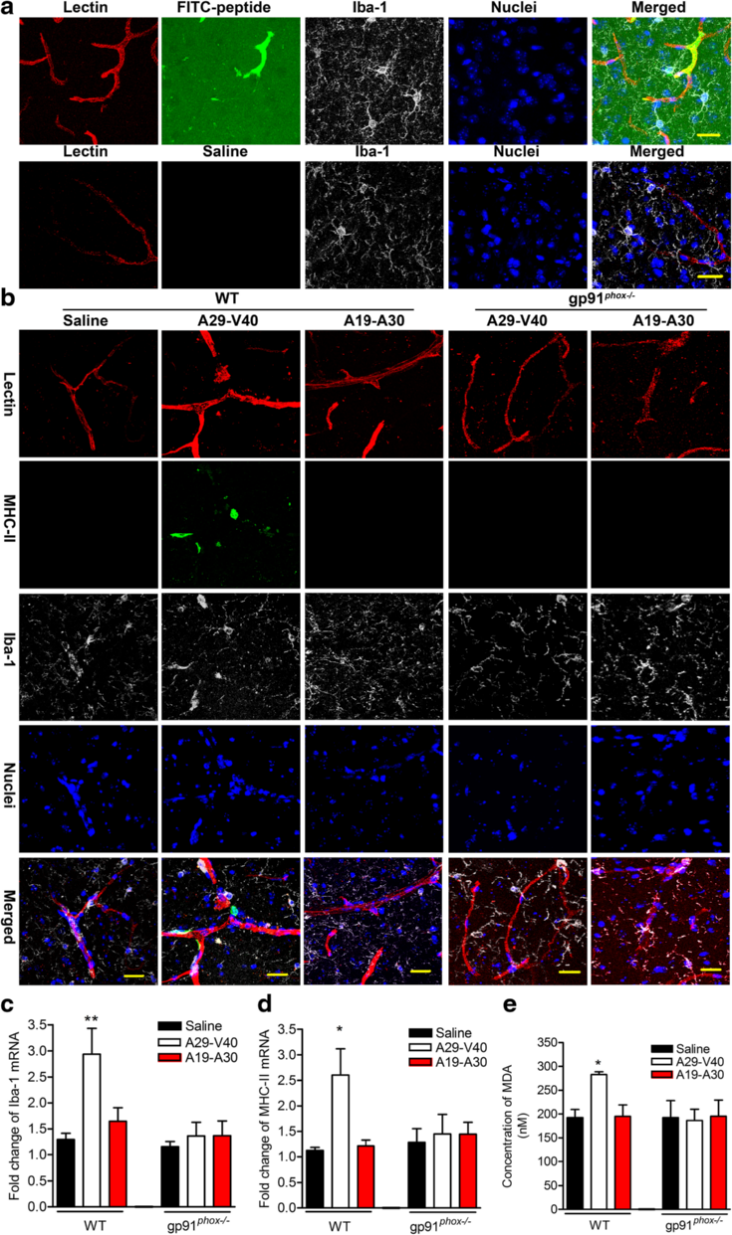
A29-V40 peptide activates microglia and promotes oxidation reaction in vivo. **a** Representative three-dimensional (3D) images showed that the peptides tested can pass through the BBB and diffuse into the interstitial tissues in the brain. Mice were i.v. injected with saline or FITC-labeled peptides (either A19-A30 or A29-V40) along with Texas red-conjugated lectin. Six hours later, animals were sacrificed and the brain tissues were stained for Ibl-1 and nuclei and imaged by a confocal microscope for 3D-reconstruction. **b** Expression of MHC-II in brain microglia. WT or gp91^*phox−/−*^ mice were i.v. injected with saline or peptides (either A19-A30 or A29-V40) along with Texas red-conjugated lectin. Twenty-four hours later, animals were sacrificed and the brain tissues were stained for Ibl-1, MHC-II, and nuclei and imaged by a confocal microscope. 3D images were reconstructed. **c** Changes in the level of Ibl-1 mRNA in mouse brains. Tissues were collected from WT or gp91^*phox−/−*^ mice injected with saline or peptides (either A19-A30 or A29-V40) for 24 h. RT-qPCR was performed by using mouse Ibl-1-specific primers and assay kits. **d** Changes in the level of MHC-II in brain tissues which were collected from WT or gp91^*phox−/−*^ mice injected with saline or peptides (either A19-A30 or A29-V40) for 24 h. RT-qPCR was performed by using mouse MHC-II-specific primers and assay kits. **e** Changes in the content of MDA in brain tissues which were collected from WT or gp91^*phox−/−*^ mice injected with saline or peptides (either A19-A30 or A29-V40) for 24 h. Assays were performed by following the manufacture manual. In **c**–**e**, ANOVA analyses followed by Newman-Keuls multiple comparison tests were performed. *n* = 3–5; *P < 0.05 compared with the corresponding saline-treated controls. *Bar* = 20 μm

Next, we examined whether A29-V40 activates microglia in vivo. Six hours after injection (i.v.) of FITC-labeled A29-V40 along with Texas red-conjugated lectin (an endothelial cell marker), the green fluorescent signals in the interstitial tissues of mouse brains were significantly increased compared to saline injection (Fig. 7a). This observation suggests that the A29-V40 peptide can pass across the BBB and diffuse into the interstitium, where it can stimulate microglia. MHC-II is a cell surface marker expressed by activated, but not resting, microglia. In our study, microglia in WT rather than in gp91^*phox−/−*^ mice, especially those surrounding the capillaries, became immunopositive for MHC-II 24 h after injection of A29-V40 peptide, but not saline or A19-A30 peptide, indicating that A29-V40 peptide activates microglia (Fig. 7b). This finding is consistent with the RT-qPCR analysis which showed higher levels of both MHC-II and Ibl-1 mRNA in the brains collected from A29-V40 peptide-injected but not A19-A30 peptide-injected WT mice. In contrast, neither A29-V40 peptide nor A19-A30 peptide could cause changes in levels of MHC-II and Ibl-1 mRNA in gp91^*phox−/−*^ brains (Fig. 7c, d). Moreover, administration of A29-V40 peptide to WT rather than to gp91^*phox−/−*^ brains increased the amount of MDA, one of the products during lipid peroxidation [33], indicating that enhanced redox reactions occurred in these brains (Fig. 7e). Taken together, our data suggest that the A29-V40 peptide is capable of activating microglia and eliciting redox reactions in vivo in a gp91^*phox*^-dependent manner.

## Discussion

α-Syn, particularly in its oligomeric form, can damage neurons and therefore plays a critical role in the pathogenesis of synucleinopathies, including PD and dementia with Lewy bodies. Exploring the mechanism by which α-Syn forms aggregates has demonstrated that the apolipoprotein-binding domain clearly contributes to its aggregation and associated toxicity. Furthermore, studies including ours have shown that a single amino acid substitution in this region, e.g., at alanine 30, which is associated with the development and progression of familial-PD [34], activates microglia more potently, exacerbating the α-Syn aggregate-induced neuronal damage [16, 35]. These findings prompted us to test the capability of a series of synthesized peptides, starting from those which cover the alanine 30 locus, to induce microglial superoxide production and neuronal damage. Our study revealed that a specific peptide, A29-V40, is capable of directly promoting the release of superoxide by microglia and inducing dopaminergic neuronal damage via a unique mechanism that is different from that caused by FL-α-Syn aggregates.

The pursuit began with comparing WT FL-α-Syn with that containing the A30P substitution, based on the relationship of this mutant form with familial PD, and the finding that FL-α-Syn featuring this change forms aggregates more quickly and becomes more toxic to neurons compared with the WT form. In our initial experiments, examining the neurotoxicity of peptides covering the alanine 30 locus, only A29-V40 was identified as initiating specific neurotoxic effects. However, when comparing the effects of the same peptides with or without the A30P alteration, the WT and mutant forms of the A29-V40 peptide were similar in terms of their toxicity to neurons. Because the effects of both the A30P and WT peptides were quite similar (Fig. 2), further experiments identifying microglia as the cells mediating the toxic effects on neurons focused on the WT peptide. Therefore, it is theoretically possible that the A30P peptide may induce a greater effect on astrocytes, while the WT peptide induces toxicity primarily through microglia, as observed (Fig. 4). However, this outcome would require a reciprocal decrease in microglial sensitivity to A30P, balancing to an equal toxicity to neurons (as in Fig. 2), a mechanism probably less likely than the proposed model of a similar, microglia-mediated response to both peptides. Nonetheless, further effects on astrocytes are an area of interest for future studies. An interesting implication of the similar microglial response to both WT and A30P peptides is that it suggests that distinct mechanisms are responsible for the neurotoxicity induced by FL-α-Syn aggregates and the peptide A29-V40. In this regard, it has been suggested that extracellular FL-α-Syn aggregates may activate microglia to generate inflammatory mediators, such as TNF-α and superoxide, to induce neuronal damage, a response that is mediated by the binding of FL-α-Syn aggregates to microglial surface receptors such as TLR2 and CD11b [3, 17]. It is possible that FL-α-Syn aggregates formed by mutant α-Syn monomers (e.g., A30P) create a conformation that is distinct from that of WT FL-α-Syn aggregates, resulting in an altered binding affinity of FL-α-Syn aggregates for their microglial receptors and allows mutant FL-α-Syn aggregates to more effectively activate microglia. In contrast, the peptides used here include only twelve amino acids, and whether they recapitulate the folded structures of the equivalent regions of the full-length protein, or represent structures with potentially very different affinities from FL-α-Syn is not known.

Two obvious questions remain to be answered. First, does this specific peptide exist in vivo? Clearly, the direct relevance of this particular peptide and its interaction with microglia to PD depends on its existence (and relative abundance) in normal and diseased human brain. However, even if the exact peptide is not present in vivo, it can be hypothesized that similar peptides generated as intermediate metabolites during the degradation of α-Syn aggregates could behave similarly to A29-V40 peptide. The rationale behind this argument is that, in theory, α-Syn aggregates are foreign materials to the immune cells, and foreign proteins are typically cleaved into peptides when recognized as antigens by the antigen processing cells (e.g., microglia and macrophages), which usually contain around nine amino acids [36], and the newly generated peptides, along with MHC-I molecule, would be further presented to T cell receptors [37]. Indeed, a series of autoantibodies against α-Syn [38] and T cells, including both CD4^+^ and CD8^+^ phenotypes, have been found in the bodily fluids and the brain tissues, respectively, of patients with PD [39–42].

The second question centers on what precisely mediates the direct interaction between the peptide and Nox2 enzyme. In this regard, it has been reported that some small peptides (e.g., substance P) [28] or small compounds (e.g., rotenone) [29] seem to preferentially bind to gp91^*phox*^, the catalytic unit of Nox2, while activating Nox2 to produce superoxide. Our data demonstrating that A29-V40-induced dopaminergic neuronal damage depends on the presence of gp91^*phox*^ in microglia and that A29-V40 is capable of precipitating gp91^*phox*^ indicate that gp91^*phox*^ might also act as a receptor for this α-Syn peptide. Clearly, in future investigations, direct binding of the peptide with gp91^*phox*^ needs to be demonstrated by more advanced technology, e.g., mass spectrometry mapping of critical amino acid sequence needed for Nox2 activation after crosslinking the peptide with gp91^*phox*^. However, this process is quite challenging because the purified gp91^*phox*^ that maintains its physiologically functional structure is currently unavailable.

**Fig. 8:**
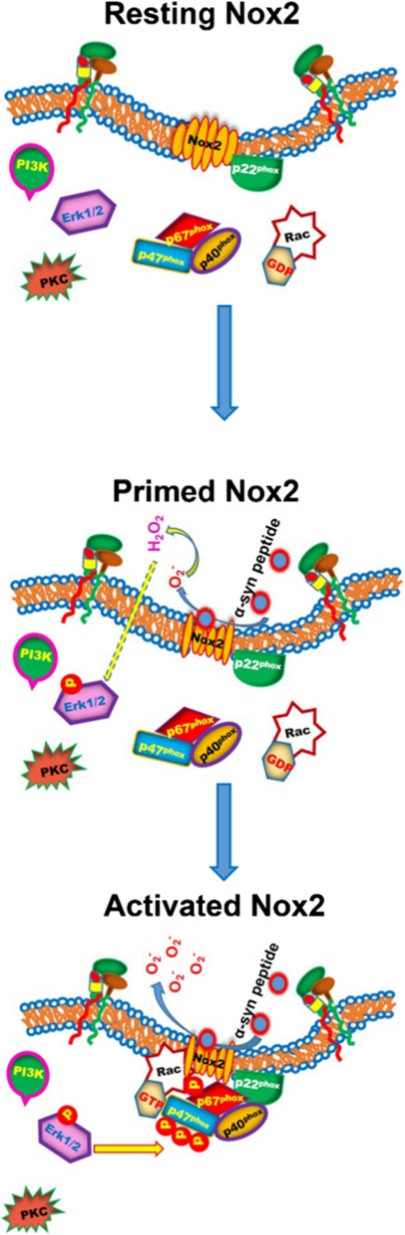
A schematic of the mechanisms by which A29-V40 peptide activates Nox2. The Nox2 catalytic unit gp91^*phox*^ is located on cell membranes together with the p22^*phox*^ unit. In a resting status, gp91^*phox*^ retains an inactivated conformation, and other components of Nox2 including p47^*phox*^, p67^*phox*^, p40^*phox*^, and small GTPase Rac stay in the cytosol. When exposed to the A29-V40 α-Syn peptide (or similar peptides with critical amino acid sequence), microglial gp91^*phox*^ is bound to this peptide, which possibly initiates a conformational change in gp91^*phox*^ and directly primes Nox2 to produce O_2_^• −^. Superoxide (O_2_^• −^) is readily converted to H_2_O_2_, either spontaneously or catalyzed by SOD. As a cell membrane-permeable chemical, H_2_O_2_ is able to diffuse into cytosol, oxidizing cysteine residues in the protein kinase Src and activating it. This process further activates the Ras-Raf-MEK-ERK signal transduction pathway to phosphorylate p47^*phox*^ and p67^*phox*^. As a result, Nox2 activity is further enhanced and more superoxide ions are produced, establishing a positive feedback loop to amplify Nox2 activation. This response includes (1) an “outside to inside” step which begins with the binding of A29-V40 α-Syn peptide and gp91^*phox*^ and leads to activation of the Ras-Raf-MEK-ERK signal transduction pathway; (2) an “inside to outside” step which is referred to Erk1/2-induced p47^*phox*^ and p67^*phox*^ phosphorylation and the ensuing amplified Nox2 activation

The other significant observation of the current investigation is that we uncovered a novel signaling pathway by which the A29-V40 peptide might activate microglia. That is, A29-V40 triggered Nox2 to generate superoxide and its metabolite H_2_O_2_ by binding to the catalytic unit gp91^*phox*^; diffusing into cytosol, H_2_O_2_ activated Erk1/2 kinase to phosphorylate p47^*phox*^ and further activated Nox2, establishing a positive feedback loop to amplify the Nox2-mediated response (Fig. 8). This signaling pathway contrasts explicitly to the previously reported pathway through which FL-α-Syn aggregates activate microglial Nox2 to induce dopaminergic neuronal damage. Specifically, FL-α-Syn aggregates bind to their microglial surface receptors such as CD11b [17] and activate several kinases (e.g., PI3K) [43], which represents an “outside-in” process during microglial activation, to phosphorylate Nox2 subunits including p47^*phox*^ and p67^*phox*^. Upon phosphorylation, these molecules are recruited from the cytosol to the cell membranes and combined with gp91^*phox*^ and p22^*phox*^ to assemble an active enzymatic complex, Nox2, and produce superoxide, which is the “inside-out” step involved in microglial activation. In the signal pathway hinted by this study, the binding of A29-V40 directly primes gp91^*phox*^ to produce superoxide, possibly via initiating changes in the conformation of gp91^*phox*^. The immediate metabolic product of superoxide (O_2_^• −^), H_2_O_2_, acts as a membrane permeable messenger to transduce the extracellular signal to the cytosol [17] by activating Erk1/2 kinase, an “outside-in” step which is present in A29-V40 peptide-induced microglial activation. Activation of Erk1/2 kinase causes the phosphorylation of Nox2 subunits including p47^*phox*^ and p67^*phox*^, and as a result, Nox2 is fully activated, which represents an outside-in step in the A29-V40-induced microglial activation. The role of H_2_O_2_ in causing full activation of Nox2 was confirmed by our data showing that timely removal of H_2_O_2_ from microglial cultures by pretreating them with SOD, an enzyme that is capable of readily converting superoxide (O_2_^• −^) to H_2_O_2_, along with catalase, another enzyme that efficiently degrades H_2_O_2_ to H_2_O and O_2_, attenuated A29-V40 peptide-induced p47^*phox*^ phosphorylation, which is a prerequisite for Nox2 activation; directly adding exogenous H_2_O_2_ to microglial cultures caused p47^*phox*^ phosphorylation even in the absence of A29-V40. Moreover, unlike PMA, an activator of PKC, which is capable of entering the cytosol by freely crossing plasma membranes and causing p47^*phox*^ phosphorylation via an unknown pathway, V29-V40 peptide failed to phosphorylate p47^*phox*^ in the presence of Erk1/2 inhibitor, indicating that this kinase is the upstream molecule of p47^*phox*^ phosphorylation. Regarding the activation of Erk1/2 by H_2_O_2_, it has been reported that this compound can oxidize the cysteine residues [44] of numerous tyrosine kinases, including Src [45], to activate them, and as a result, activated Src kinase leads to a cascade of reactions in which Ras-Raf-MEK-Erk1/2 are involved [46].

The biological significance of microglial activation is of course neurodegeneration. In in vitro assays, the V29-V40 peptide exhibits toxicity to neurons via activating microglia to produce superoxide. Utilizing this peptide to in vivo models, we also found that it could activate microglia and stimulate the oxidation reaction. However, whether this peptide can damage dopaminergic neurons in vivo remains unknown, which warrants further investigation. Furthermore, besides the A29-V40 peptide, in future investigations, it would be interesting to examine whether specific neurotoxic peptides also exist in the regions that correspond to the glutamic acid 46- or the alanine 53-containing locus.

## Conclusions

In summary, we identified the α-Syn peptide, A29-V40, as a neuronal toxicant that is capable of directly inducing the release of microglial superoxide leading to dopaminergic neurodegeneration. These findings provide novel mechanistic information about how α-Syn may cause neuronal injury, possibly via the abnormal metabolites of α-Syn aggregates.

## Abbreviations

A30P, alanine 30 to proline-mutated α-synuclein; A53T, alanine 53 to threonine-mutated α-synuclein; ANOVA, analysis of variance; Apo, apocynin; BBB, blood-brain barrier; CNS, central nervous system; DA, dopamine; DAPI, 4′,6-diamidino-2-phenylindole, dihydrochloride; DMEM, Dulbecco’s modified Eagle medium; FL-α-Syn, full-length α-synuclein; HBSS, Hank’s balanced salt solution; LPS, lipopolysaccharide; MDA, malondialdehyde; MHC-II, major histocompatibility complex II; NAC, non-amyloid-β component; Nox2, NADPH oxidase; PBS, phosphate-buffered saline; PD, Parkinson’s disease; PMA, phorbol 12-myristate 13-acetate; ROS, reactive oxygen species; SOD, superoxide dismutase; TH, tyrosine hydroxylase; TNF-α, tumor necrosis factor α; WT, wild type; α-Syn, α-synuclein

## References

[1] Maries E, Dass B, Collier TJ, Kordower JH, Steece-Collier K (2003). The role of alpha-synuclein in Parkinson’s disease: insights from animal models. Nat Rev Neurosci.

[2] Irwin DJ, Lee VM, Trojanowski JQ (2013). Parkinson’s disease dementia: convergence of alpha-synuclein, tau and amyloid-beta pathologies. Nat Rev Neurosci.

[3] Kim C, Ho DH, Suk JE, You S, Michael S, Kang J, Joong Lee S, Masliah E, Hwang D, Lee HJ (2013). Neuron-released oligomeric alpha-synuclein is an endogenous agonist of TLR2 for paracrine activation of microglia. Nat Commun.

[4] Zhang W, Wang T, Pei Z, Miller DS, Wu X, Block ML, Wilson B, Zhang W, Zhou Y, Hong JS (2005). Aggregated alpha-synuclein activates microglia: a process leading to disease progression in Parkinson’s disease. FASEB J.

[5] Lee HJ, Bae EJ, Lee SJ (2014). Extracellular alpha—synuclein-a novel and crucial factor in Lewy body diseases. Nat Rev Neurol.

[6] Glass CK, Saijo K, Winner B, Marchetto MC, Gage FH (2010). Mechanisms underlying inflammation in neurodegeneration. Cell.

[7] Ueda K, Fukushima H, Masliah E, Xia Y, Iwai A, Yoshimoto M, Otero DA, Kondo J, Ihara Y, Saitoh T (1993). Molecular cloning of cDNA encoding an unrecognized component of amyloid in Alzheimer disease. Proc Natl Acad Sci U S A.

[8] Bartels T, Choi JG, Selkoe DJ (2011). Alpha-synuclein occurs physiologically as a helically folded tetramer that resists aggregation. Nature.

[9] Burre J, Sharma M, Tsetsenis T, Buchman V, Etherton MR, Sudhof TC (2010). Alpha-synuclein promotes SNARE-complex assembly in vivo and in vitro. Science.

[10] Giasson BI, Murray IV, Trojanowski JQ, Lee VM (2001). A hydrophobic stretch of 12 amino acid residues in the middle of alpha-synuclein is essential for filament assembly. J Biol Chem.

[11] Wood SJ, Wypych J, Steavenson S, Louis JC, Citron M, Biere AL (1999). alpha-synuclein fibrillogenesis is nucleation-dependent. Implications for the pathogenesis of Parkinson’s disease. J Biol Chem.

[12] Bisaglia M, Trolio A, Bellanda M, Bergantino E, Bubacco L, Mammi S (2006). Structure and topology of the non-amyloid-beta component fragment of human alpha-synuclein bound to micelles: implications for the aggregation process. Protein Sci.

[13] Li J, Uversky VN, Fink AL (2002). Conformational behavior of human alpha-synuclein is modulated by familial Parkinson’s disease point mutations A30P and A53T. Neurotoxicol.

[14] Li J, Uversky VN, Fink AL (2001). Effect of familial Parkinson’s disease point mutations A30P and A53T on the structural properties, aggregation, and fibrillation of human alpha-synuclein. Biochem.

[15] Conway KA, Lee SJ, Rochet JC, Ding TT, Williamson RE, Lansbury PT (2000). Acceleration of oligomerization, not fibrillization, is a shared property of both alpha-synuclein mutations linked to early-onset Parkinson’s disease: implications for pathogenesis and therapy. Proc Natl Acad Sci U S A.

[16] Zhang W, Dallas S, Zhang D, Guo JP, Pang H, Wilson B, Miller DS, Chen B, Zhang W, McGeer PL (2007). Microglial PHOX and Mac-1 are essential to the enhanced dopaminergic neurodegeneration elicited by A30P and A53T mutant alpha-synuclein. Glia.

[17] Wang S, Chu CH, Stewart T, Ginghina C, Wang Y, Nie H, Guo M, Wilson B, Hong JS, Zhang J (2015). Alpha-synuclein, a chemoattractant, directs microglial migration via H2O2-dependent Lyn phosphorylation. Proc Natl Acad Sci U S A.

[18] Chen SH, Oyarzabal EA, Hong JS (2013). Preparation of rodent primary cultures for neuron-glia, mixed glia, enriched microglia, and reconstituted cultures with microglia. Methods Mol Biol.

[19] Tan AS, Berridge MV (2000). Superoxide produced by activated neutrophils efficiently reduces the tetrazolium salt, WST-1 to produce a soluble formazan: a simple colorimetric assay for measuring respiratory burst activation and for screening anti-inflammatory agents. J Immunol Methods.

[20] Wang S, Cao C, Chen Z, Bankaitis V, Tzima E, Sheibani N, Burridge K (2012). Pericytes regulate vascular basement membrane remodeling and govern neutrophil extravasation during inflammation. PLoS One.

[21] Qian L, Wei SJ, Zhang D, Hu X, Xu Z, Wilson B, El-Benna J, Hong JS, Flood PM (2008). Potent anti-inflammatory and neuroprotective effects of TGF-beta1 are mediated through the inhibition of ERK and p47phox-Ser345 phosphorylation and translocation in microglia. J Immunol.

[22] Taschenberger G, Garrido M, Tereshchenko Y, Bahr M, Zweckstetter M, Kugler S (2012). Aggregation of alphaSynuclein promotes progressive in vivo neurotoxicity in adult rat dopaminergic neurons. Acta Neuropathol.

[23] Friedman L, Mytilineou C (1987). The toxicity of MPTP to dopamine neurons in culture is reduced at high concentrations. Neurosci Lett.

[24] Casper D, Mytilineou C, Blum M (1991). EGF enhances the survival of dopamine neurons in rat embryonic mesencephalon primary cell culture. J Neurosci Res.

[25] Bronstein DM, Perez-Otano I, Sun V, Mullis Sawin SB, Chan J, Wu GC, Hudson PM, Kong LY, Hong JS, McMillian MK (1995). Glia-dependent neurotoxicity and neuroprotection in mesencephalic cultures. Brain Res.

[26] Hines DJ, Choi HB, Hines RM, Phillips AG, MacVicar BA (2013). Prevention of LPS-induced microglia activation, cytokine production and sickness behavior with TLR4 receptor interfering peptides. PLoS One.

[27] Raad H, Paclet MH, Boussetta T, Kroviarski Y, Morel F, Quinn MT, Gougerot-Pocidalo MA, Dang PM, El-Benna J (2009). Regulation of the phagocyte NADPH oxidase activity: phosphorylation of gp91phox/NOX2 by protein kinase C enhances its diaphorase activity and binding to Rac2, p67phox, and p47phox. FASEB J.

[28] Wang Q, Chu CH, Qian L, Chen SH, Wilson B, Oyarzabal E, Jiang L, Ali S, Robinson B, Kim HC (2014). Substance P exacerbates dopaminergic neurodegeneration through neurokinin-1 receptor-independent activation of microglial NADPH oxidase. J Neurosci.

[29] Zhou H, Zhang F, Chen SH, Zhang D, Wilson B, Hong JS, Gao HM (2012). Rotenone activates phagocyte NADPH oxidase by binding to its membrane subunit gp91phox. Free Radic Biol Med.

[30] Bedard K, Krause KH (2007). The NOX family of ROS-generating NADPH oxidases: physiology and pathophysiology. Physiol Rev.

[31] Dewas C, Fay M, Gougerot-Pocidalo MA, El-Benna J (2000). The mitogen-activated protein kinase extracellular signal-regulated kinase 1/2 pathway is involved in formyl-methionyl-leucyl-phenylalanine-induced p47phox phosphorylation in human neutrophils. J Immunol.

[32] Namura S, Iihara K, Takami S, Nagata I, Kikuchi H, Matsushita K, Moskowitz MA, Bonventre JV, Alessandrini A (2001). Intravenous administration of MEK inhibitor U0126 affords brain protection against forebrain ischemia and focal cerebral ischemia. Proc Natl Acad Sci U S A.

[33] Nielsen F, Mikkelsen BB, Nielsen JB, Andersen HR, Grandjean P (1997). Plasma malondialdehyde as biomarker for oxidative stress: reference interval and effects of life-style factors. Clin Chem.

[34] Kruger R, Kuhn W, Muller T, Woitalla D, Graeber M, Kosel S, Przuntek H, Epplen JT, Schols L, Riess O (1998). Ala30Pro mutation in the gene encoding alpha-synuclein in Parkinson’s disease. Nat Genet.

[35] Lashuel HA, Overk CR, Oueslati A, Masliah E (2013). The many faces of alpha-synuclein: from structure and toxicity to therapeutic target. Nat Rev Neurosci.

[36] Kloetzel PM (2001). Antigen processing by the proteasome. Nat Rev Mol Cell Biol.

[37] Deleidi M, Maetzler W (2012). Protein clearance mechanisms of alpha-synuclein and amyloid-Beta in lewy body disorders. Int J Alzheimer’s Dis.

[38] Smith LM, Schiess MC, Coffey MP, Klaver AC, Loeffler DA (2012). Alpha-synuclein and anti-alpha-synuclein antibodies in Parkinson’s disease, atypical Parkinson syndromes, REM sleep behavior disorder, and healthy controls. PLoS One.

[39] Appel SH (2009). CD4+ T cells mediate cytotoxicity in neurodegenerative diseases. J Clin Invest.

[40] Brochard V, Combadiere B, Prigent A, Laouar Y, Perrin A, Beray-Berthat V, Bonduelle O, Alvarez-Fischer D, Callebert J, Launay JM (2009). Infiltration of CD4+ lymphocytes into the brain contributes to neurodegeneration in a mouse model of Parkinson disease. J Clin Invest.

[41] Cebrian C, Zucca FA, Mauri P, Steinbeck JA, Studer L, Scherzer CR, Kanter E, Budhu S, Mandelbaum J, Vonsattel JP (2014). MHC-I expression renders catecholaminergic neurons susceptible to T-cell-mediated degeneration. Nat Commun.

[42] Mosley RL, Hutter-Saunders JA, Stone DK, Gendelman HE (2012). Inflammation and adaptive immunity in Parkinson’s disease. Cold Spring Harbor Perspect Med.

[43] Zhang D, Hu X, Qian L, Chen SH, Zhou H, Wilson B, Miller DS, Hong JS (2011). Microglial MAC1 receptor and PI3K are essential in mediating beta-amyloid peptide-induced microglial activation and subsequent neurotoxicity. J Neuroinflammation.

[44] Burgoyne JR, Madhani M, Cuello F, Charles RL, Brennan JP, Schroder E, Browning DD, Eaton P (2007). Cysteine redox sensor in PKGIa enables oxidant-induced activation. Science.

[45] Kemble DJ, Sun G (2009). Direct and specific inactivation of protein tyrosine kinases in the Src and FGFR families by reversible cysteine oxidation. Proc Natl Acad Sci U S A.

[46] Chang F, Steelman LS, Lee JT, Shelton JG, Navolanic PM, Blalock WL, Franklin RA, McCubrey JA (2003). Signal transduction mediated by the Ras/Raf/MEK/ERK pathway from cytokine receptors to transcription factors: potential targeting for therapeutic intervention. Leukemia.

